# Postnatal maturation of the basolateral and paralaminar amygdala in rodents: implications for social–emotional development

**DOI:** 10.3389/fnins.2026.1787955

**Published:** 2026-03-20

**Authors:** David Saxon, Joshua G. Corbin

**Affiliations:** 1 Georgetown University Medical Center, Washington, DC, United States; 2 Center for Neuroscience Research, Children's Research Institute, Children's National Hospital, Washington, DC, United States

**Keywords:** adolescent brain development, basolateral amygdala, paralaminar amygdala, postnatal maturation, social–emotional development

## Abstract

Across vertebrate species, the amygdala is a central node of the social brain network, an interconnected set of nuclei for regulation of innate and learned social and emotional behaviors. The amygdala undergoes significant structural and functional changes from juvenile to adult stages of life, driving the emergence of complex social and emotional behaviors. Focusing primarily on rodent animal models, in this review we describe the dramatic changes that unfold from juvenile stages to young adulthood in two amygdala nuclei that undergo significant post-natal growth, the basolateral amygdala (BLA) and paralaminar nucleus (PL). Interestingly, while both the BLA and PL mature during post-natal stages, neurons in the BLA mature earlier than the PL. The differing maturational rates along with differences in input/output connectivity of these nuclei indicate that they play complementary roles in the emergence of amygdala-driven social–emotional behaviors, such as responses to fear, social hierarchical behaviors and responses to novel cues. The post-natal development of the BLA, and likely the PL, which is less well studied, is highly shaped by the environment, particularly the quality of early caregiving. Understanding the developmental processes that unfold during this critical period of life and their sensitivity to environmental disruption will be key to developing biologically informed interventional strategies for a host of human disorders characterized by neuro-atypical social–emotional behaviors.

## Introduction

**Figure 1:**
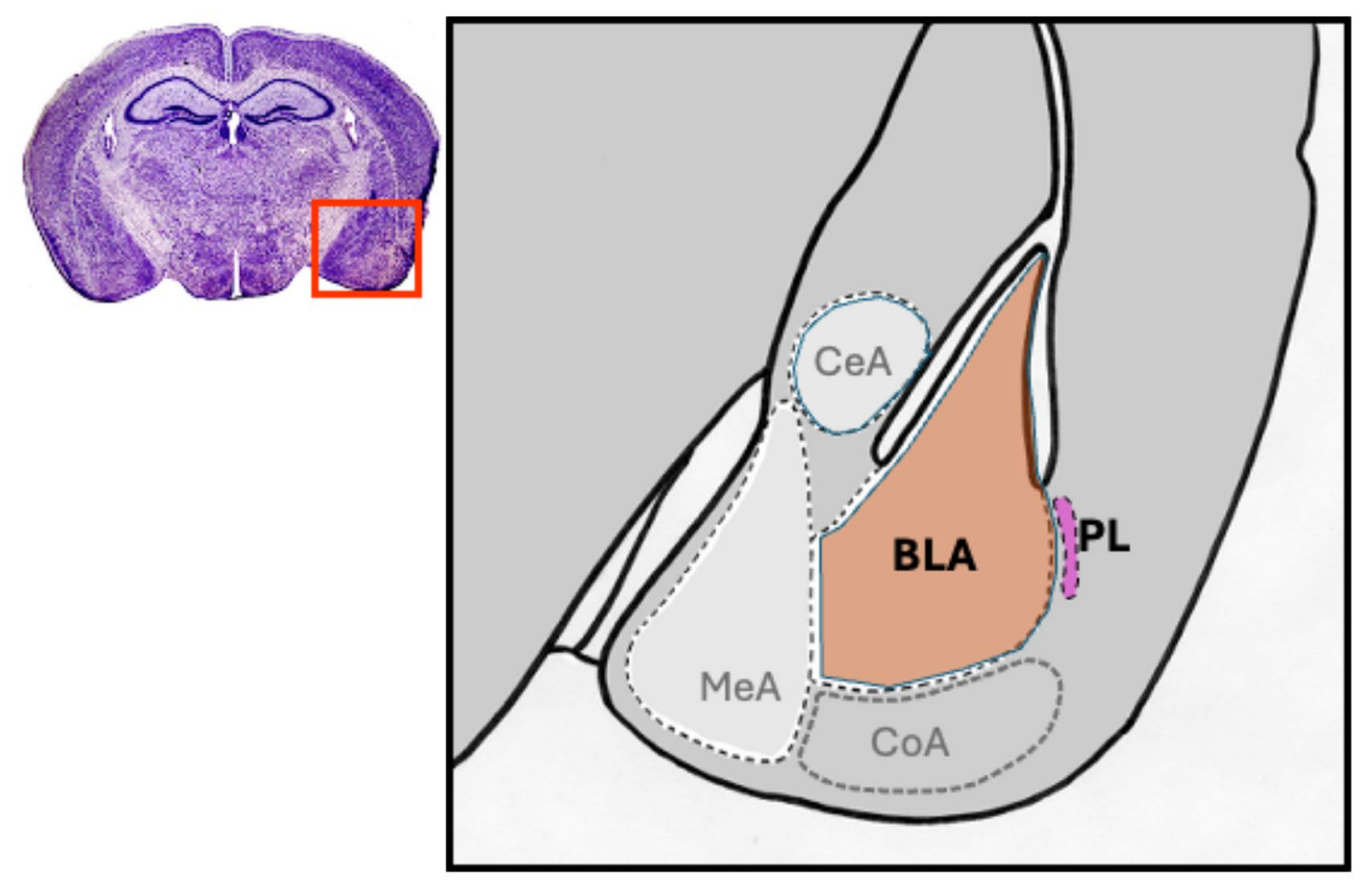
Anatomy the major amygdala showing major nuclei and highlighting the location of the basolateral (BLA, light brown) and paralaminar (PL, pink) nuclei in the mouse in coronal view. Like in larger mammals, the PL is adjacent to the BLA. Abbreviations: CeA central amygdala, CoA cortical amygdala, MeA medial amygdala.

The amygdala is a key brain region involved in integrating environmental stimuli for appropriate behavioral outputs related to social navigation and avoidance of threats. Importantly, the amygdala undergoes remarkable post-natal growth which is critical for the behavioral transition from the juvenile period to adulthood. The amygdala comprises approximately 10–12 distinct nuclei that perform different roles in social–emotional behavior (Aerts and Seuntjens, 2021; Adolphs, 2010; Newman, 1999; Phelps and LeDoux, 2005). Of these nuclei, the basolateral (BLA) and paralaminar (PL) amygdala nuclei appear to uniquely display structural and functional changes between juvenile and adult ages. Anatomically, in rodents these nuclei abut each other in the ventral telencephalon (Figure 1), yet are distinct in their neuronal composition, connectivity and as we describe in detail below, their maturational trajectories. While the postnatal development of the BLA is well-characterized in rodents, the PL has thus far been almost exclusively studied in larger mammals, including humans, non-human primates, cats, sheep, and tree shrews (Avino et al., 2018; Bernier et al., 2002; Braak and Braak, 1983; Caroline Crosby and Humphrey, 1939; Chareyron et al., 2016; de Campo et al., 2017; Page et al., 2022; Piumatti et al., 2018; Sah et al., 2003; Sorrells et al., 2019; Yachnis et al., 2000). However, the recent identification of the PL in the mouse has opened the opportunity for mechanistic studies in a genetically tractable model, as well as comparisons to the BLA (Alderman et al., 2024). In this review, we describe the maturational trajectories of the rodent BLA and PL from juvenile to adult ages, focusing on hallmark changes in molecular expression, morphology, electrophysiological properties, and synaptic connectivity. We conclude by discussing how these developmental processes are affected by external stressors in the rodent model resulting in atypical behaviors.

**Figure 2:**
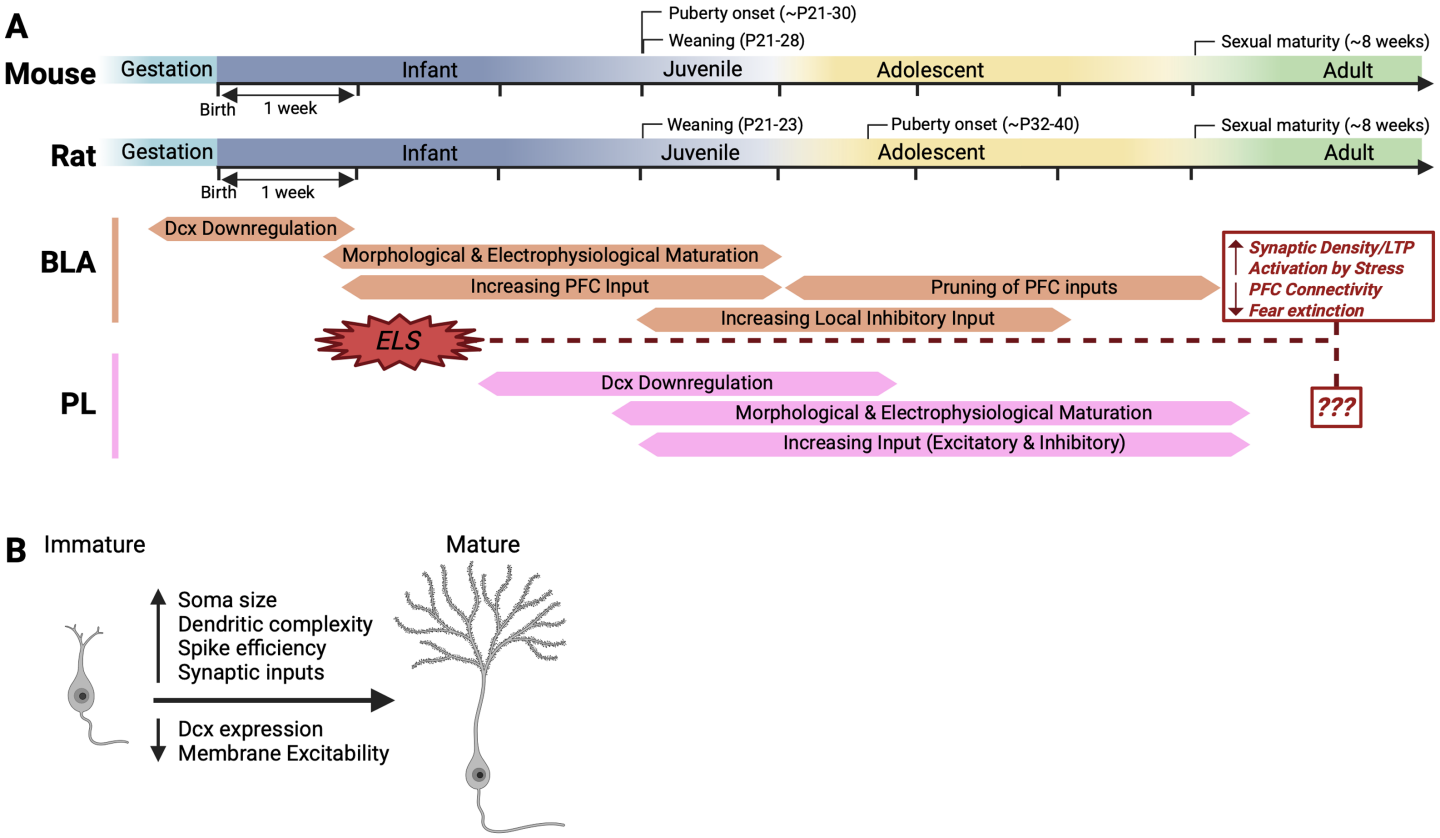
Timelines and benchmarks of BLA and PL maturation in mouse and rat. **(A)** Timelines of the major changes during maturation of BLA and PL principal neurons, and the consequences of early life stress (ELS) on BLA maturation. **(B)** Major characteristics of neuronal maturation. Immature neurons display a simple, migratory morphology and lack elaborated dendrites and elongated axons. Mature neurons have large cell bodies with complex dendritic trees and ramified axons, display abundant synaptic connectivity, no longer express migratory proteins, and exhibit electrophysiological properties that promote efficient input–output coding. Created in https://BioRender.com.

In mammals, brain development begins gestationally and continues into post-natal life, with major changes in growth and connectivity of neurons into early adulthood. The post-natal period from birth to adulthood is divided into infancy, juvenile and adolescent stages. In humans the juvenile period is from about age two until ten with adolescence defined as the period from the onset of sexual maturation, which varies from individual to individual, from about age ten until age nineteen (Bell, 2018). In rodents, the juvenile period is approximately from weaning at three weeks until about four weeks, with adolescence occurring between about the fourth to the sixth postnatal week (Schneider, 2013) (Figure 2). However, in both rodents and humans, the onset and completion of adolescence are not defined by clear timepoints. In addition to the onset and progression of puberty adolescence is characterized by numerous and dramatic behavioral changes occurring before and after the pubertal window. These include increased social independence, pursuit of novelty and risk, the enhanced ability to navigate a complex social environment, and maturation of emotional processing (Juraska and Willing, 2017; Schneider, 2013; Spear, 2000). Across mammalian species, these changes support a shift from caregiver bonding and exploratory social behaviors in juvenile ages, to complex affiliative, aversive, and defensive behaviors in adolescence and adulthood (Ferrara and Opendak, 2023; Harda et al., 2025; Harlow and Zimmermann, 1959; Lloyd et al., 2023; Meaney and Stewart, 1981; Panksepp, 1981; Sullivan et al., 2000). This transition is linked to the changing needs of the individual for survival, which at early ages are tied to the primary caregiver, but later require independent detection of resources and threats.

The overwhelming majority of neurons in the mammalian brain are born during gestation, after which they undergo several developmental steps, culminating in reaching their full maturational state upon completion of adolescence (Figure 2). Neuronal maturation is defined as the process whereby a postmitotic neuron undergoes changes in molecular, morphological, electrophysiological, and synaptic connectivity, which subsequently remain relatively stable, equipping the neuron to reliably process stimuli and drive behaviors within dedicated circuits. Early to mid-gestation is characterized by extensive proliferation of neural precursors. Once neurons exit the cell cycle, they undergo both long- and short-range migration to their final destinations. The period of late gestation to early infancy is characterized by the elaboration of dendrites and axons, myelination and the formation of nascent synapses (Zeiss, 2021). Juvenile and adolescent periods are characterized by modeling and strengthening of neuronal connectivity, processes of which underly behavioral transitions. Each of these steps of development are identified by distinct molecular, morphological and electrophysiological readouts (Figure 2). For example, migrating immature postmitotic neurons in the developing brain are defined by their expression of doublecortin (Dcx) and polysialyated cell adhesion molecule (Psa-Ncam) (Gleeson et al., 1999; Ono et al., 1994), and at this stage of their lifecycle lack axons and dendrites and are generally devoid of synaptic connectivity. Once settling in their final locations, neurons undergo extensive changes in morphological complexity, including a growth in soma size and the ramification of dendritic trees (Hoshiba et al., 2016; Wise et al., 1979). Along with these morphological changes, there is a reduction in membrane excitability and an increase in the speed, amplitude, and frequency of action potentials, which result in more effective input–output coding (Gao and Ziskind-Conhaim, 1998; Nikitin et al., 2017; Schulz, 2006). Finally, the establishment and refinement of synaptic connections is seen from both local interneurons and long-range input from projection neurons from other brain regions, which can be observed at the structural and electrophysiological levels as stronger and more frequent inhibitory and excitatory post synaptic currents. These connections then undergo pruning and reorganization as a neuron adopts more specific roles in maturing circuits (Harrill et al., 2015; Ichikawa et al., 1993).

### Neuronal maturation in the postnatal BLA and PL

Principal output neurons in both the BLA and PL are excitatory (Alderman et al., 2024; McDonald, 2020). However, the trajectory of neuronal maturation differs between the two nuclei (Figure 2). Electrophysiological and morphological maturation in the BLA occurs much earlier than the PL, primarily during the first postnatal month. From P7-28, BLA neurons in the rat increase in soma size and dendritic complexity, and display a reduction in membrane excitability driven by decreased input resistance and time constant (Ehrlich et al., 2012; Ryan et al., 2016). During this same window, action potentials become faster, maximum firing frequency increases, and neurons begin to exhibit burst firing. From P28 to later ages (>P35) in the rat, BLA principal neurons do not show significant electrophysiological changes, and are therefore considered physiologically mature (Ehrlich et al., 2012). Interestingly, during this stage there is an emergence of sex differences in electrophysiological and morphological properties; action potentials become slightly smaller and faster in males, with increased excitability at adulthood compared to females, while females undergo a greater change in spine morphology (Guily et al., 2022).

Unlike BLA neurons, which are largely mature by the end of the first postnatal month, PL neurons are still in early stages of maturation through juvenile ages. In the mouse, from P7-28, expression of Dcx is approximately 100-fold more dense in the PL than in the BLA; though Dcx expression is not specific to neurons, the majority (65–80%) of Dcx + cells in the PL co-express the neuronal marker NeuN (Alderman et al., 2024). From P21-28, a substantial number of mouse PL neurons continue to express Dcx. These Dcx-expressing neurons show interesting heterogeneity: some feature a small soma, lack dendrites, and exhibit immature electrophysiological features including high input resistance and slow, low-amplitude action potentials, while others show more developed dendritic trees and spike properties despite retaining Dcx expression. Mouse PL neurons that no longer express Dcx at P21-28 display more developed morphological and electrophysiological profiles, such as decreased excitability, faster and higher amplitude action potential spikes, and hallmark features of synaptic connectivity at both the structural and electrophysiological levels (Alderman et al., 2024). From juvenile ages (P21-28) to early adulthood (P60), majority of mouse PL neurons remain in the PL; no neurons are lost to cell death, but interestingly a small population migrates to the nearby ventral endopiriform (VEn) and a trace number (<10%) maintain Dcx expression into adulthood (Alderman et al., 2024). From P59-78, mouse PL neurons express much lower membrane excitability, greater amplitude, speed, and frequency of action potentials, highly complex dendritic trees, and increased input/output connectivity compared to P21-28 (Alderman et al., 2024; Saxon et al., 2024).

Therefore, maturation of molecular, morphological and electrophysiological properties is more protracted in the PL compared to the BLA, continuing through adolescence. Upon reaching maturity, the PL and BLA both exhibit electrophysiological heterogeneity, containing projection neurons with bursting and delayed-spiking phenotypes (Rainnie et al., 1993; Sah et al., 2003; Saxon et al., 2024; Washburn and Moises, 1992), which is a broadly conserved feature of excitatory projection neurons across the brain (Agmon and Connors, 1992; Chen et al., 2022; Connors and Gutnick, 1990; Kasper et al., 1994; Sun et al., 2013; Suzuki and Bekkers, 2006). Importantly, however, mature PL neurons are distinct from their BLA counterparts, exhibiting distinctive electrophysiological and morphological properties (Ehrlich et al., 2012; Guily et al., 2022; Saxon et al., 2024), as well as different connectivity (discussed below).

### Changes in inputs to the BLA and PL

The BLA receives major inputs from prefrontal, insular, visual, auditory and olfactory cortices, the hippocampus, and thalamus, as well as from other amygdala nuclei (Hintiryan et al., 2021; McDonald, 2020, 1998). Interestingly, the number of postnatal changes in these BLA inputs is region-specific. From P7-19 in the rat BLA, inputs from the thalamus and substantia innominata are stable, and inputs from the auditory cortex increase only slightly. However, the greatest increase in inputs comes from the prefrontal cortex (PFC), where BLA- projecting neurons are almost absent at P7 but increase in number, density, and distribution from P9-19 (Bouwmeester et al., 2002). Alongside the significant increase in PFC neuronal projections to the BLA, afferent tracing from the PFC shows that the density of their fibers in the BLA increases starting as early as P10, spreading from the medial to lateral aspect and peaking at P30 (Arruda-Carvalho et al., 2017). This is coupled with electrophysiological strengthening of these PFC-BLA connections from P15-30 both presynaptically (decreased paired-pulse ratio) and postsynaptically (increased AMPA/NMDA ratio) (Arruda-Carvalho et al., 2017). Following this growth of input connectivity, PFC inputs are pruned: after P30, PFC fiber density in the BLA drops (Arruda-Carvalho et al., 2017), and the number of neurons projecting from the PFC decreases substantially from P45 to 90 (Cressman et al., 2010). Thus, postnatal maturation of the BLA is characterized by significant strengthening of inputs specifically from the PFC peaking at the beginning of adolescence, followed by synaptic pruning through adolescence. Parallel to these changes in long range input from the PFC to the BLA, there are changes in inhibitory inputs from local interneurons to the BLA. GABA responses in the rat BLA shift from depolarizing to hyperpolarizing around P21 (Ehrlich et al., 2013). This postnatal GABA shift, a critical event in the transition from spontaneous to sensory-driven synaptic plasticity (Peerboom and Wierenga, 2021; Toyoizumi et al., 2013), occurs later in the BLA than the neocortex and hippocampus, where it occurs before P14 (Rivera et al., 1999; Tyzio et al., 2007). Following this GABA shift, feedforward inhibition from the PFC increases dramatically from P21 to P30, and inhibitory signaling to BLA neurons continues strengthening through adolescence until adult ages (Arruda-Carvalho et al., 2017; Selleck et al., 2018).

In the mouse, inputs to the PL emanate from other amygdala nuclei, including the BLA and cortical amygdala, as well as from multiple regions of the olfactory, insular, piriform, and entorhinal cortices (Saxon et al., 2024). While some of these inputs overlap with the BLA, the PL is more closely connected with the main olfactory network. During juvenile ages (P21-28), immature Dcx-expressing PL neurons display sparse spontaneous synaptic activity, suggesting that inputs remain immature alongside other neuronal properties. In contrast, synaptic responses at adult ages (P59-78) are of higher frequency and amplitude, particularly in inhibitory synaptic currents which are nearly absent at P21-28 (Alderman et al., 2024; Saxon et al., 2024). This indicates that, like in the BLA, synaptic inputs are dynamic during postnatal PL development, with PL input connectivity maturing on a delayed timeline compared to BLA neurons. The delayed development of PL inputs during adolescence, as well as its connectivity with olfactory systems, suggests different roles for these nuclei in navigating the changing environment from juvenile ages through adolescence.

### Postnatal changes in the amygdala support social and emotional development from juvenile ages to adulthood

In infancy, when maternal care is required for survival, innate preference for a caregiver is hard-wired (Harlow and Zimmermann, 1959; Hess, 1959). For example, newborn rodents are unable to detect danger: in the first postnatal week conditioned odors universally produce affiliative responses, even those that predict painful stimuli (Sullivan et al., 2000). As the BLA develops during the second postnatal week, it supports the emergence of fear conditioning (Camp and Rudy, 1988; Haroutunian and Campbell, 1979; Thompson et al., 2008). This coincides with locomotion in juvenile rodents, and may equip animals to better detect danger during bouts of independent exploration (Altman and Sudarshan, 1975; Bolles and Woods, 1964). Interestingly, at these ages the presence of the maternal caregiver suppresses fear conditioning, likely through reduced glucocorticoid activation of the BLA (Moriceau and Sullivan, 2006). Thus, in infancy the BLA facilitates the delicate balance between affiliation with a caregiver, which is critical immediately after birth, and the recognition of danger, which becomes important with increased independence at juvenile ages. Thus, as discussed below, deficient or low-quality maternal care during early infancy has lasting consequences both on the developing amygdala and social behaviors.

As rodents approach weaning age (~P21), efficient processing of novel cues, both social and non-social, is necessary for navigating an unpredictable environment free from a caregiver. At this age, the BLA enables the processing of novel affiliative and aversive cues, both with conspecifics as well as the broader environment. Fear conditioning behaviors become robust around P21-24, including trace fear conditioning and fear extinction (Barnet and Hunt, 2005; Burman et al., 2014; Kim and Richardson, 2008). The BLA is strongly activated during fear expression at this age (Deal et al., 2016), before synaptic pruning of PFC afferents and increased inhibitory activity develops during adolescence as discussed above (Arruda-Carvalho et al., 2017; Ehrlich et al., 2012; Selleck et al., 2018). Animals at weaning age also begin to display more complex social behaviors, including affiliative play and territorial behaviors (Meaney and Stewart, 1981; Panksepp, 1981), which continue to develop in adolescence and endow animals the ability to navigate their place in the social hierarchy (Burghardt, 2001; Panksepp, 1998). While the brain networks contributing to these social behaviors are widely distributed, both the PFC and BLA are implicated. The PFC may play a role in adequate discrimination of social partners and receptiveness to play-fighting during development (Bell et al., 2009; Pellis et al., 1992). The adolescent BLA, on the other hand, is necessary for social drive (Ferrara et al., 2021), and dopaminergic activity in the BLA modulates approach-avoidance decisions toward novel peers (Opendak et al., 2021).

Much less is known about the behavioral role of the PL. However, a recent study examined *c-fos* expression in the mouse PL in response to novel and predator odorants (Alderman et al., 2024). From juvenile ages (P25-27) to adulthood (P60-100), PL neurons acquired a responsiveness to novel non-predator odors. These results suggest that as PL neurons mature, they may acquire a role in processing salient non-threatening cues. This would contrast with the earlier maturing BLA, which is more dedicated to the needs of the animal for survival, changing from early care-giver dependence to discrimination of threatening cues as the animal becomes independent. However, mechanistic studies linking PL neuronal maturation to specific behaviors are still lacking, and the functional role of the PL remains an open question for future investigation.

### Postnatal BLA and PL development is sensitive to altered environments

The postnatal development of the amygdala is greatly influenced by the social environment. During preweaning ages, the most well-known environmental factor is the quality of parental care. Cellular and circuit development of the BLA occurs before weaning, when rodent pups are almost entirely dependent on the mother for survival. Thus, even small variations in maternal care have lasting effects on BLA architecture. For example, in adult rats the density of GABA and benzodiazepine receptors in the BLA is predicted by the quality of maternal care received during infancy (Caldji et al., 2003, 1998). To test the effects of inadequate maternal care as a model of early life stress (ELS) in rodents, one widely used paradigm is the limitation of nesting available to the nursing dam. Mouse and rat pups raised in this limited nesting environment display abnormal behaviors into adolescence and adulthood, including anxiety and depressive-like behaviors, cognitive dysfunction and social deficits (Chen and Baram, 2016). Alongside these behavioral abnormalities, many changes in BLA structure and function are seen in this model. After a limited nesting environment from P1-9, male rats display increased BLA dendrite complexity and spine density by P20, followed by increased LTP and perineuronal net density at P22-28 (Guadagno et al., 2018b, 2020). Further, both male and female rats display reduced functional connectivity between the BLA and PFC into adulthood (P74-76), alongside impaired fear extinction (Guadagno et al., 2018a). Limited bedding from P2-21 increases anxiety behaviors and BLA activity from P21-68 during a stress-induced hyperphagia paradigm (Malter Cohen et al., 2013). Rats raised with limited bedding from P8-12 display reduced social interaction at P20 and P45, as well as depressive-like behavior at P45 in the forced swim test that is correlated with increased *c-fos* activity in the amygdala, including the basal and lateral nuclei, and rescued by chemical inhibition broadly targeting the amygdala (Raineki et al., 2012). Overall, inadequate maternal care produces changes in the BLA, characterized by increased neuronal sensitivity, enhanced synaptic plasticity, and altered connectivity with the PFC. These alterations emerge by weaning age, when fear conditioning and social behaviors are beginning to peak, and contributes to the social and emotional dysfunctions seen into adulthood.

In contrast, the effects of ELS on PL development are less well studied. However, to date a single study in primates suggests a dramatic effect. In this study, a week of maternal deprivation at one week or one month of age reduced the expression of the excitatory marker *tbr1* in the PL at three months of age, suggesting a defect in gene expression, a decrease in the number of mature PL excitatory neurons or excessive excitatory activity (de Campo et al., 2017). This suggests that the postnatal development of the PL is closely shaped by, and subsequently influences, early social behaviors, presenting a testable hypothesis for future studies. Regardless of the exact role of the PL, the adolescent time frame of neuronal maturation makes it well placed to be susceptible to environmental insults during this critical phase, especially stress-inducing paradigms.

## Discussion

The postnatal development of the amygdala is characterized by structural and functional transformations that coincide with the behavioral transition from juvenile ages to adulthood with these changes occurring primarily in the BLA and PL nuclei. In rodents, the BLA undergoes rapid maturation during juvenile ages, supporting the emergence of fear learning and early social behaviors. In parallel, the PL undergoes a different maturational trajectory characterized by neurons that are highly immature by the beginning of juvenile stages. Although less explored than the BLA, the PL potentially contributes to the refinement of the discrimination of novel cues during this period. The BLA is highly sensitive to environmental input, particularly the quality of early caregiving, with disruptions producing lasting circuit abnormalities and social–emotional behavioral disruptions in animal models. The PL is likely also sensitive to disrupted care environments, though further work is needed to evaluate this relationship. Future research into how the BLA and PL individually and collectively drive social–emotional changes from juvenile ages to adulthood and how these processes are disrupted in both environmental and genetic models of neurodevelopmental and behavioral disorders, will be essential for the prevention, evaluation, and treatment of a host of human conditions.
